# Improving the technical efficiency of public health centers in Cambodia: a two-stage data envelopment analysis

**DOI:** 10.1186/s12913-023-09570-w

**Published:** 2023-08-28

**Authors:** Dominik Beiter, Sokunthea Koy, Steffen Flessa

**Affiliations:** 1 Social Health Protection Programme, Deutsche Gesellschaft für Internationale Zusammenarbeit (GIZ), Phnom Penh, Cambodia; 2 Department of General Business Administration and Health Care Management, University of Greifswald, Greifswald, Germany

**Keywords:** Data envelopment analysis, Efficiency, Health centers, Cambodia

## Abstract

**Background:**

Cambodia is undergoing a series of reforms with the objective of reaching universal health coverage. Information on the causes of inefficiencies in health facilities could pave the way for a better utilization of limited resources available to ensure the best possible health care for the population.

**Objectives:**

The purpose of this study is to evaluate the technical efficiency of health centers and the determinants for inefficiencies.

**Methods:**

This cross-sectional study used secondary data from a costing study on 43 health centers in six Cambodian provinces (2016–2017). Firstly, the Data Envelopment Analysis method with output-orientation was applied to calculate efficiency scores by selecting multiple input and output variables. Secondly, a tobit regression was performed to analyze potential explanatory variables that could influence the inefficiency of health centers.

**Results:**

Study findings showed that 18 (43%) health centers were operating inefficiently with reference to the variable returns to scale efficiency frontier and had a mean pure technical efficiency score of 0.87. Overall, 22 (51%) revealed deficits in producing outputs at an optimal scale size. Distance to the next referral hospital, size and quality performance of the health centers were significantly correlated with health center inefficiencies.

**Conclusion:**

Differences in efficiency exist among health centers in Cambodia. Inefficient health centers can improve their technical efficiency by increasing the utilization and quality of health services, even if it involves higher costs. Technical efficiency should be continuously monitored to observe changes in health center performance over time.

## Highlights

• A total of 41% of the sample health centers were identified as technically inefficient with the potential to increase their output capacity.

• With the given financial resources of health centers, a 13%-increase in service delivery would be possible.

• Substantial efficiency gains could be generated by improved utilization and increased quality of health services.

• Technical efficiency of health centers in Cambodia will improve if quality of services, organizational and health center management are strengthened.

## Background

Cambodian health policy has focused strongly on implementing health sector reforms, including its financing mechanisms, to improve the quality and reliability of healthcare provision, and to develop human resource capacity to achieve universal health coverage (UHC) [1, 2]. The public health sector aims to ensure equitable and affordable access to healthcare for all citizens, although institutional capacity and quality of care is still in development and health care services are largely sought at private care providers [1, 3]. Out-of-pocket payments continue to be the major source of funding for healthcare, leading to further impoverishment, inequity, and restricted access to health care services [2, 4].

Nevertheless, considerable progress has been made, particularly in the delivery and uptake of preventive services. However, little attention has been paid to the cost of health services and its utilization in relation to financial and capital inputs. More recently, the country’s public health strategy aims to increase the cost-effectiveness of health services in order to finance public health facilities without relying on donor contributions [2]. Simultaneously, Cambodia is transitioning to a new health financing system with the launch of its 2016–2025 Social Protection Policy Framework, which aims to protect poor and vulnerable groups by building the infrastructure necessary for ensuring efficiency and social sustainability in the future. Also, the recent SARS-CoV-2-pandemic has been a stress test for public finances, revealing the importance of resilient health financing systems and crisis preparedness against rapid disease transmission [5].

As financial and healthcare staff resources are scarce, inefficiency in the provision of healthcare services can lead to or result from misallocations [6]. The World Health Organization estimates that on average 20 to 40% of national health expenditure could be saved through efficiency gains, achieved by reallocating resources, health workforce, infrastructure and health services according to the actual needs [7, 8].

In economic theory, production units that produce maximum output with a limited set of given inputs are defined as operating on the maximum production frontier line, also described as "technically efficient" [9, 10]. When applying microeconomic production theory considerations, a “production process” of a healthcare facility (unit) can be divided into input and output categories [11]. Potential inefficiencies of a healthcare facility can then be assessed by analyzing the use of inputs to produce the output. In this way, inefficiencies in healthcare facilities can be identified and corrected through better use of available (limited) resources, ultimately to provide the best possible healthcare to the population [8, 12].

This study aims to evaluate the technical efficiency of 43 health centers in Cambodia and the determinants for inefficiencies. Technical efficiency describes the ability of a production unit to maximize its output when only a certain amount of available input resources are available [13]. We wanted to understand if there are differences in technical efficiency among health centers in Cambodia, to estimate the magnitudes of output increases and/or input reductions that would have been required to make relatively inefficient health centers efficient, and to identify which factors could be a predictor for preventable inefficiencies. We also wanted to understand, if there are differences in technical efficiency between two types of health centers: health centers ‘with’ and ‘without’ beds.

To our knowledge, no study has yet addressed the question whether or not health centers in Cambodia operate at optimal scale levels (the ability to generate the most outputs per input), nor has relative productivity on the health center level been evaluated [14–17]. A recent study to assess technical efficiency of public health services in Cambodia at the provincial level confirmed the underutilization of health services [17].

In recent decades, a considerable amount of literature on benchmarking and efficiency assessment techniques such as Data Envelopment Analysis (DEA) has been published to shed light on the different levels of health sector performance [9, 14, 18–23]. DEA method was chosen because it is best suited to estimate the technical efficiency of health centers, which typically use multiple inputs (for example budget or human resources) to produce multiple outputs. By calculating efficiency scores for each health center it is possible to make relative comparisons with the other units in the sample.

However, DEA literature on individual primary care health center performance is limited and has been conducted mainly in sub-Saharan African countries rather than in the Southeast Asian region [20, 24–28].

## Methods

### Study setting

Cambodia has an estimated population size of 15.6 million and is classified as a lower-middle-income country [29, 30]. About 14% of the population live below the national poverty line [29]. The national public health system is made up of health centers (with and without beds), and referral, provincial and national hospitals, categorized according to their functional complexity. The public primary sector focuses on the management of communicable diseases and maternal and child health in the impoverished population [31]. Health centers without beds consist of 10–12 healthcare workers covering an area of 5,000 to 20,000 people [3, 32]. They provide initial consultations and primary diagnosis, emergency first aid, chronic disease care, maternal and child care, immunization, health education and referrals [3]. Health centers with beds have larger departments and supplementary medical equipment, but generally include a similar set of health services plus limited inpatient care. In this study, we focused on health centers, with and without beds, within the public sector. To allow comparison between the two different types of health centers in the sample, we excluded costing data on inpatient services.

### Study design and data collection

This cross-sectional study used secondary costing data collected by Jacobs et al. [33] during 2016–17, to conduct a two stage DEA efficiency evaluation model. The study sample consisted of 43 health centers. Health centers belonged to six provinces: Kampong Thom, Kampot, Kampong Chhnang, Kampong Cham, Takeo, and Battambang. Only one health center with beds was selected per province. Costing and price data were collected in USD (2017) currency, using a fixed exchange rate of KHR4,000 to US$1. The methodology to collect costing information has been described elsewhere Jacobs et al. [33]. Health center quality score data was gathered for the whole sample from the Cambodian Health Quality Improvement Project [34]. For the first stage of the DEA analysis, a data set for all health centers was compiled to build the main input and output variables, including the expenditure sections of personnel, assets, medical stores, buildings, and health center production costs. For the second stage analysis, additional spatial data was retrieved from the open data website “Open Development Cambodia” [35].

### Data analysis

Firstly, the DEA method with output-orientation was applied to calculate technical efficiency scores by selecting multiple input and output variables. Secondly, a tobit regression was performed to analyze potential explanatory variables that could influence the inefficiency of health centers.

### DEA framework

DEA is a non-parametric linear programming method for evaluating the relative technical efficiency in economics and applies Farrell’s production theory [13] on technical efficiency of production units, also called decision-making units (DMUs), using the benchmarking of constant and variable returns to scale as an optimization method [14, 36]. In this study, DMUs are health center units under study in the first stage analysis.

**Fig. 1:**
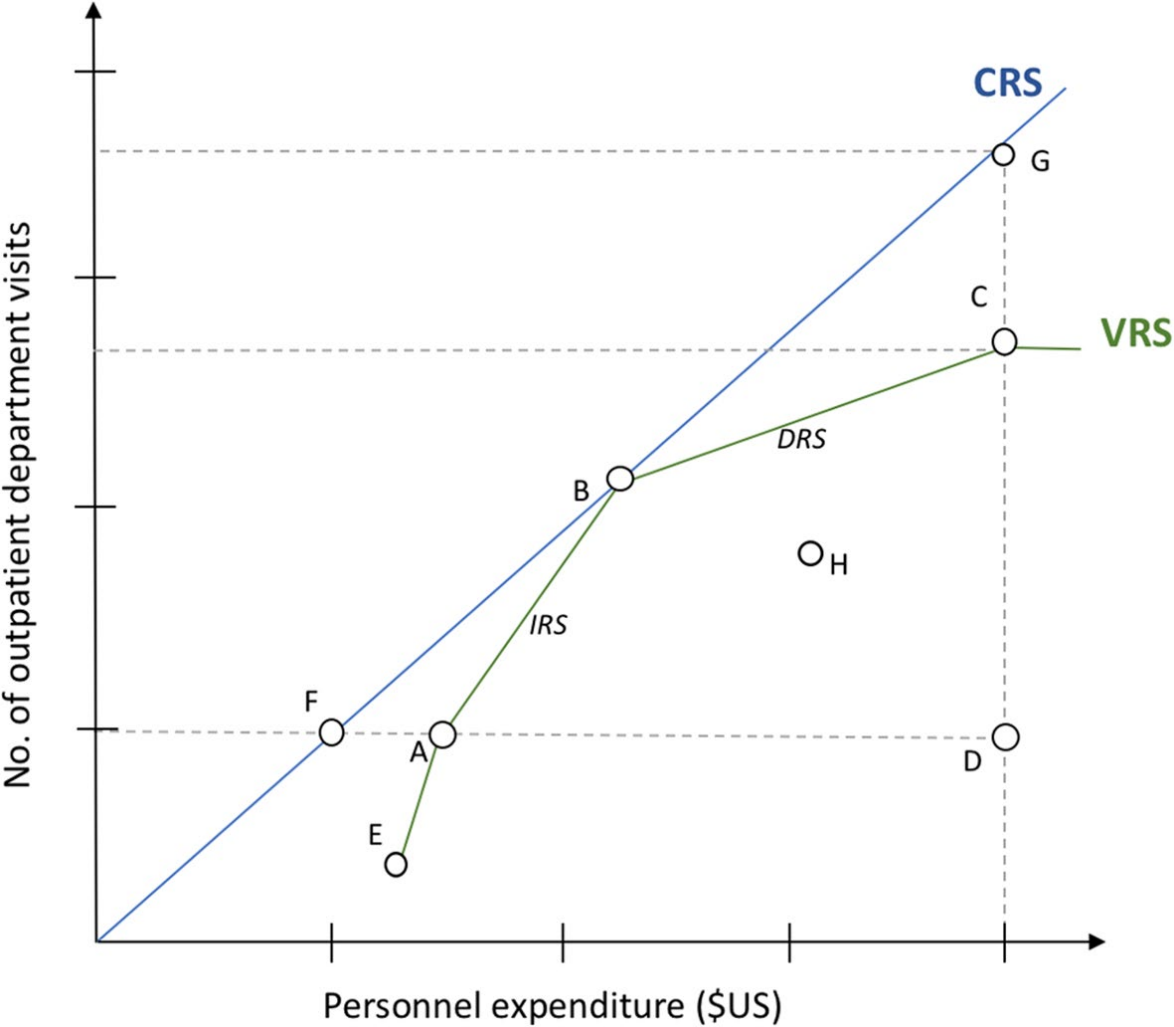
Figure describes two technical efficiency production frontier models for health centers, namely with constant returns to scale (CRS) and variable returns to scale (VRS) assumption. Most productive scale size is in point B. X-axis: Input-variable 'personnel expenditure (USD)', Y-axis: Output variable 'Number of outpatient department visits'

DEA measures technical efficiency using economies of scale assumptions, either constant returns to scale (CRS) or variable returns to scale (VRS). In CRS models, the optimal mix of inputs and outputs for the production efficiency frontier line calculation is anticipated independently of the economies of scale of operation [24]. Overall, technical efficiency for each sample health center is measured with reference to the best practice DMUs, with the objective to achieve optimal technical efficiency [36]. VRS models estimate the “pure” technical efficiency, assuming that not all health centers operate at optimal scale [37]. Central to the VRS model assumption is that health centers operate either on increasing, constant, or decreasing return to scale levels [38]. When a health center indicates increasing returns to scale (IRS), a one percent rise on the input will be followed by more than a percent rise in output performance. In contrast, a health center operating on decreasing returns to scale (DRS), would exhibit congestion with a lower proportionate increase of one percent in the outputs, caused by a one percent rise in the input resources, as shown in the example of point C in Fig. 1.

### First stage

Output-orientation was applied, which states that DEA model calculations are guided by how much additional outputs can be produced if the input side remains constant because a health center can better control its inputs than its outputs [14, 39]. In the Cambodian case, input categories are fixed with labor, medical supplies (stores) and other infrastructural costs, as those are largely allocated centrally by the Ministry of Health. Health center managers have no control over the size and scope of the health centers they manage, which is why output orientation was ultimately chosen.

According to Charnes et al. [40] technical efficiency of a DMU is defined as the maximum of a ratio of the aggregate weighted outputs against the aggregate of its weighted inputs, under the condition that the similar rates for each DMU (health center) are less than or equal to 1, expressed in the following term:

$$\mathrm{Technical}\;\mathrm{Efficiency}=\left(\frac{Weighted\;sum\;of\;health\;center\;outputs}{Weighted\;sum\;of\;health\;center\;inputs}\right)$$

Any health center scoring 100% is regarded as technically efficient and a score below 100% is regarded as technically inefficient. The following DEA model term determined weights $\mu$_r_ for outputs and $\nu$_i_ for inputs entirely from the input and output data of those DMUs in the peer group, so as to maximize the efficiency rating of a health center being evaluated [38]. DEA efficiency computation with multiple inputs and outputs for *j* health centers is assumed as follows:

$$Maximize\;\theta_o=\frac{\sum_{r=1}^s\mu_ry_{ro}}{\sum_{i=1}^mv_ix_{io}}$$

$$subject\;to\frac{\sum_{r=1}^s\mu_ry_{rj}}{\sum_{i=1}^mv_ix_{ij}}\leq1 \; \; \; \; \; u_{r},v_i\geq0\;for\;all\;r\;and\;i.$$

The optimal set of weights is represented with $\mu$_r_ for output and $\nu$_i_ for input. y_rj_ is the amount of output *r* and the amount of input *i* for the DMU *j.* Each health center is using inputs *x*_*ij*_ to produce a set of health center outputs *y*_*rj*_. The relative efficiency score of a given health center (${\theta }_{o}$) can be acquired by resolving the output-oriented CRS linear programming model following Charnes et al. [40].

To test for the robustness of the DEA technical efficiency measures, we conducted jackknife analysis method following Jehu-Appiah et al. [25] to assess extreme outliers by omitting one observation at a time. The efficiency scores obtained were robust as indicated by Spearman rank correlation coefficient, which was very close to one. Accordingly, we also omitted all six health centers with beds and retained all other 37 health centers without beds to observe any major changes in the DEA technical efficiency measures.

For each health center in our sample, the scale efficiency (SE) value is calculated, which indicates the “(…) loss from not operating at an optimal scale size.” [37].

$${SE}_{k}=\frac{{\theta }^{CRS}}{{\theta }^{VRS}}$$

Scale efficiency is a measure describing “(…) the ability to get the most outputs per input” [37] by comparing an average product of a health center “X” to average product at the technically optimal point (see also Fig. 1). A scale efficiency value below 1 (lower than SE_k_ = 1 (100%)) explains how much a DMU could expand its output or input (in percent) until it reaches optimal scale size (SE_k_ = 1 (100%)) relative to the CRS frontier line [38].

To determine what amounts of input reductions and/or output increases would be required to make the individual technically-inefficient health center efficient, DEA-generated weights of each of the health centers in the efficiency reference set used to identify an individual health center’s inefficiency must be multiplied by the actual outputs and inputs of each reference health center [41]. After multiplying the output and input values with the DEA-generated weights, the slack values are summed, and the results then compared to the health center's outputs and inputs. Following Ozcan [38], target input values are calculated by subtracting the slack value. Target outputs are then calculated by multiplying the efficiency score and adding an output-specific slack value, where applicable.

Efficient targets for inputs and outputs in the output oriented VRS model were calculated as follows:

$$\begin{array}{cc}\mathrm{Inputs}: {x}_{io}^{\mathrm{^{\prime}}}={x}_{io}- {s}_{i}^{-*}& i=1, \dots m\end{array}$$

$$\begin{array}{cc}\mathrm{Outputs}: {\mathrm{y}}_{ro}^{^{\prime}}={\phi }^{*}{y}_{ro}+{s}_{i}^{+*}& r=1, \dots s.\end{array}$$

### First stage dataset

We selected input and output variables based on previous research conducted on this subject [9, 14, 16] and on a robustness evaluation following Valdmanis [42] variety testing of alternative input–output variables. Consequently, we selected the following variables:

- Inputs
  - ◦ Personnel cost of health center: cost of staff per annum in US$
  - ◦ Medical stores: cost for drugs, medical materials and vaccine per annum in US$
  - ◦ Other expenditure: cost of all other resources (depreciation of equipment, vehicles and buildings; all other running expenditure) per annum in US$
  - ◦ Health center size: Measurement of size of health center in m^2^
- Outputs
  - ◦ Number of outpatient department visits: Number of visits in the general consultation department per annum
  - ◦ Number of other patient contacts: Number of visits in all other departments (maternity, family planning, prevention, chronic diseases) per annum
  - ◦ Quality score of health center: Overall quality score of each health center for diagnoses and treatment according to national standards (%)

These variables represent the inputs and outputs as main components of the efficiency formula given above. It should be noted that salaries and wages for staff of public healthcare facilities are fixed by the government. Consequently, they are the same for all health facilities. Differences in personnel cost represent different numbers and/or qualifications of staff. Equally, drugs, medical materials and vaccines are bought from the central medical store (CMS), a government institution in the capital Phnom Penh, at fixed prices. Consequently, differing costs are due to differing quantity and quality of materials. For instance, the frequency of antibiotics prescribed differs between public health centers as a major component of technical efficiency.

### Second stage

In a second stage, we analyzed whether health centers had some common characteristics that indicated potential causes for inefficiency or a better utilization of resources. A tobit regression was performed to determine whether potential geographical, institutional and health center-specific factors could be associated with the (in-)efficiency scores of both constant and variable returns to scale that were calculated in the first stage [43–45]. The tobit regression model solves the normalization and truncation issue by censoring the lower and upper bounds [38].

Therefore, before running the tobit regression model, the efficiency score value $\theta$ had to be converted to an inefficiency score $\widehat{\theta }$ to obtain a continuous, left-censored variable at zero that can be used in a tobit regression model:

$$Transformed\;Inefficiency\;score\;\widehat\theta=\left(\frac1\theta\right)-1$$

The tobit model equation is expressed as:

$${Y}_{i}= {\beta }_{0}+{\beta }_{1}{x}_{1}+{\beta }_{2}{x}_{2}+\dots +{\beta }_{n}{x}_{n}+{\varepsilon }_{i}$$

For this, we state the overall Y_*i*_ efficiency score under the Variable Returns to Scale assumption from the previous stage for every health center, x_n_ for the explanatory variables, ${\beta }_{n}$ exemplifies the unobserved tobit coefficient, and ${\varepsilon }_{i}$ the error term with the assumption of normal distribution. Prior to the second stage tobit regression, Pearson’s correlation coefficients between all variables were analyzed.

The finally selected empirical model was:

$${Y}_{i} = {\beta }_{0}+ {\beta }_{1}OPD+{\beta }_{2}DIST1+{\beta }_{3}DIST2+ {\beta }_{4}BEDS+ {\beta }_{5}POP+ {\beta }_{6}SIZE {+ \beta }_{7}QUAL +{\varepsilon }_{i}$$

### Second stage data set (predictor variables)

Table 1 provides an overview of the selected variables for a tobit regression, which were controlled for confounding. Table 2 gives a definition of studied variables. Remoteness of a health facility was approached by measuring the two distance variables ‘Distance of a health center to the nearest Provincial Hospital (km)’, and ‘Distance of health center to nearest Hospital (km)’. Hospitals in rural Cambodia often can be found in larger settlements (health operational districts), normally also providing enhanced road access and infrastructure. Distance measurements were calculated with the geographic information system software “QGIS” using spatial location data and a hub distance-command. Additional variables included were: the number of OPD visits per year, a binary variable to distinguish health center with and without beds, a variable listing the health center population coverage (number of persons covered), health center quality scores (in %) from the Health Equity and Quality Improvement Project (H-EQIP) and Health center size (in m^2^).

**Table 1:** Descriptive statistics of explanatory variables for the tobit regression models

Variable	Mean	SD	Min	Max
VRS inefficiency score variable	.095242	.2007258	0	.9039168
CRS inefficiency score variable	.219885	.4216576	0	1.941029
OPD visits per year (2016)	10,514.6	5,875.0	2,224	24,551
HC distance to closest provincial referral hospital (km)	31.2076	12.3028	4.165653	63.05392
HC distance to closest referral hospital (km)	11.01142	5.83498	.0194614	24.86173
Health centers with beds (binary)		.3506046	0	1
Health center population coverage (No. of persons)	14,099.67	4,252.45	5,678	23,644
Health center size (m^2^)	248.4651	160.2032	108	859
Quality score (%)	64.94954	15.5221	30.93	87.47

*Abbreviations: CRS* Constant returns to scale, *HC* Health center, *OPD* Outpatient department, *VRS* Variable returns to scale

**Table 2:** Definition of study variables (second stage tobit regression)

Variable definition	Definition	Measurement
**Dependent variable**		
VRS Inefficiency score (first stage calculation result)	Variable Returns to Scale Inefficiency Score	Score value (range between 0 and 1)
CRS Inefficiency score (first stage calculation result)	Constant Returns to Scale Inefficiency Score	Score value (range between 0 and 1)
**Independent variables**		
OPD visits per year (OPD)	Number of outpatient department visits per health center in 2016	Number of patient visits in 2016/2017
HC distance to closest provincial referral hospital (km) (DIST1)	Distance of health center to closest provincial referral hospital	in kilometers (km)
HC distance to closest referral hospital (km) (DIST2)	Distance of health center to closest provincial referral hospital	in kilometers (km)
Health centers with beds (binary) (BEDS)	Binary variable indicating health center with beds	1, if health center with beds
Health center population coverage (POP)	Health center population coverage	in number of persons covered
Health center size (m^2^) (SIZE)	Size of a health center	in square meters (m^2^)
Quality score (%) (QUAL)	H-EQUIP quality score	in percent (%)

STATA version 13 was used for all analyses.

## Results

### Input and output variables

The sample of 43 health centers had an annual total of 452,128 outpatient consultations (OPD) and 696,882 other contacts (see Table 3). Outputs were produced at an input cost of US$1,538,568 on staff remuneration, US$1,785,293 for medical stores (medicines, vaccines, and medical products) and US$507,389 for other expenditures. The average annual personnel cost per health center in 2016 amounted to US$35,781, with a maximum expenditure of US$72,162. In general, health centers with beds spent more on human resources, averaging US$50,694 compared to regular health centers averaging US$33,362, and were relatively large with an average size of 473 m^2^ compared to regular health centers at 212 m^2^.

**Table 3:** Descriptive statistics of input and output variables for DEA calculation of public health centers in Cambodia (*N* = 43)

Variable	Total	Mean	Minimum	Maximum	SD
**Inputs**					
Personnel cost of health center (USD)	1,538,568	35,781	20,335	72,162	12,321
Medical stores (USD)	1,785,293	41,518	11,105	84,414	17,685
Other expenditure (USD)	507,389	11,800	1,515	37,575	6,805
Health center size (m^2^)	10,684	248	108	859	160
**Outputs**					
Number of outpatient department visits in 2016	452,128	10,515	2,224	24,551	5,875
Number of other (non-OPD) patient contacts in 2016 ^a^	696,882	16,207	2,081	35,548	8,628
Quality score of health center (%)		65	31	87	15

*Abbreviations: DEA* Data envelopment analysis, *OPD* Outpatient department

^a^ Including maternity, chronic, prevention, and others

The input variable “Medical Stores” averaged US$41,518 per health center. The input variable “Other expenditure” ranged from US$1,515 to US$37,575.

### Technical efficiency

Results from the first stage DEA analysis are presented in Table 4. Comparing the results in mean and median shows that DEA efficiency values differ with respect to the selection between the overall VRS or CRS scale assumption. 25 (58%) of DMUs were operating at the VRS efficiency frontier (VRS efficient health centers), while 18 (42%) were VRS inefficient. Overall, 21 DMUs are considered as scale efficient, the remaining being scale inefficient. 16 (37%) health centers encountered increasing returns to scale and 6 (14%) decreasing returns to scale. 22 health centers (51%) revealed deficits in producing sufficient outputs from the given set of inputs at optimal scale size, equaling a 13% efficiency loss (SE = 0.87).

**Table 4:** Output oriented DEA efficiency scores with constant (CRS) and variable returns to scale (VRS) for input–output-variable mix of Table 3 descriptive statistics for health centers in Cambodia (*N* = 43)

DMUs (Health center)	Efficiency scores			Return to scale	Reference set (lambda weights)
	**CRS_TE**	**VRS_TE**	**SCALE**		
HC 1	1	1	1	0	
HC 2	1	1	1	0	
HC 3	1	1	1	0	
HC 4	1	1	1	0	
HC 5	1	1	1	0	
HC 6	0.926516	0.944097	0.981378	IRS	HC 2 (.616667); HC 13 (.110347); HC 27 (.143388)
HC 7	0.823031	1	0.823031	IRS	HC 2 (.591709)
HC 8	0.929980	0.970958	0.957796	IRS	HC 2 (.236744); HC 4(.258978); HC 27 (.350084)
HC 9	1	1	1	0	
HC 10	1	1	1	0	
HC 11	0.970534	0.972125	0.998363	IRS	HC 2 (.0049712); HC 12 (.04238); HC 13 (.118779); HC 16 (.458219); HC 27 (.334716)
HC 12	1	1	1	0	
HC 13	1	1	1	0	
HC 14	0.924828	1	0.924828	IRS	HC 2 (.571054); HC 13 (.148609)
HC 15	1	1	1	0	
HC 16	1	1	1	0	
HC 17	0.551434	0.595809	0.925521	IRS	HC 2 (.0988419); HC 3 (.0833971); HC 5 (.107221); HC 27 (.297564); HCWB 6 (.0087852)
HC 18	0.698550	0.845348	0.826347	IRS	HC 2 (.42111); HC 33 (.222267); HCWB 6 (.201971)
HC 19	0.608834	0.654597	0.930089	IRS	HC 2 (.270345); HC 13 (.0849155); HCWB 6 (.299336)
HC 20	1	1	1	0	
HC 21	0.997646	1	0.997646	IRS	HC 4(.0239438); HC 5 (.247834); HC 27 (.620256)
HC 22	0.570968	0.802447	0.711534	IRS	HC 12 (.63266); HC 27 (.170368)
HC 23	1	1	1	0	
HC 24	0.893118	0.893818	0.999217	IRS	HC 1 (.0106323); HC 27 (.876941)
HC 25	0.965437	1	0.965437	DRS	
HC 26	1	1	1	0	
HC 27	1	1	1	0	
HC 28	1	1	1	0	
HC 29	0.913396	0.975793	0.936054	IRS	HC 2 (.0628929); HC 12 (.669898); HC 13 (.102414)
HC 30	0.927664	0.969734	0.956617	DRS	HC 3 (.0774864); HC 12 (.0285018); HC 30 (.698273); HCWB 6 (.168323)
HC 31	0.830824	1	0.830824	DRS	
HC 32	0.897419	0.897419	1	0	HC 1 (.0475921); HC 5 (.0673381); HC 27 (.385181); HC 28 (.292933)
HC 33	1	1	1	0	
HC 34	0.790522	0.846834	0.933502	IRS	HC 13 (.470987); HC 36 (.0350239)
HC 35	0.474630	0.525233	0.903657	IRS	HC 2 (.185108); HC 13 (.0734652); HC 33 (.0057588); HC 36 (.0667072); HC 35 (.194193)
HC 36	1	1	1	0	
HC 37	0.808853	0.934671	0.865388	DRS	HC 13 (.125852); HC 36 (.291207); HCWB 6 (.517611)
HCWB 1	0.340017	0.658735	0.516166	IRS	HC 2 (.283871); HC 3 (.33489); HCWB 6 (.0399741)
HCWB 2	0.958440	0.969113	0.988986	IRS	HC 2 (.251167); HC 4(.146328); HC 27 (.571618);
HCWB 3	1	1	1	0	
HCWB 4	0.592387	0.951985	0.622265	DRS	HC 9 (.178858); HC 12 (.154944); HC 27 (.237126); HC 31 (.381058)
HCWB 5	0.415684	0.770264	0.539663	DRS	HC 31 (.169717); HCWB 6 (.60055)
HCWB 6	1	1	1	0	
**Min**	0.340017	0.595809	0.516166		
**Max**	1	1	1		
**Mean (all HCs)**	0.879319	0.934395	0.933356		
**Median (all HCs)**	0.970534	1	0.999217		
**SD**	0.183671	0.120931	0.122314		
**N**	43	43	43		

CRS_TE = technical efficiency from CRS DEA; VRS_TE = technical efficiency from VRS DEA; scale (scale efficiency) = CRS_TE/VRS_TE

*Abbreviations: CRS* Constant returns to scale, *DEA* data envelopment analysis, *DMUs* Decision-making units (operating units), *HC* Health center, *HCWB* Health center with beds, *VRS* Variable returns to scale

Considering the output orientation of the model and holding the input side constant, the scale inefficient DMUs could theoretically produce 13% more output. The VRS-scale-inefficient DMUs encountered scores ranging between 60 and 97% with a mean of 86%. Thirteen of the 22 scale inefficient health centers (VRS) had higher pure technical efficiency scores than their corresponding scale efficiency scores, indicating that the input sides of the DMUs were either too large or the utilization rate and quality scores on the output side were too low.

Overall, 16 (37%) health centers had increasing returns to scale while six (14%) had decreasing returns to scale (DRS) indicating that their outputs increased to a smaller extent when the input side was increased. According to DRS logics, two of the largest health centers with beds would have to reduce their input costs side to attain optimal economies of scale and move towards scale efficiency. The same scenario applies to two normal-sized health centers without beds.

Table 5 gives results for a selection of health centers (12 of 21 inefficient health centers) on how the performance of inefficient DMUs of VRS could become more efficient through targeted input reductions or output increases, weighted by efficiency reference sets. DEA outputs include an efficiency reference set with corresponding weights that enable projections from the inefficient target health centers to a composite position of overall technical efficiency by either increasing health center inputs and/or outputs.

**Table 5:** Efficiency scores, input–output original data and target projections for inefficient health centers according to VRS assumption (*n* = 12)

Health center	Score-VRS	Input/output	Actual quantity	Target quantity	Difference	Percentage
HC 17	0.595809	Personnel cost	27,821	16,576	-11,245	-40%
		Stores	33,429	19,917	-13,512	-40%
		Other expenditure	14,752	5,931	-8,821	-60%
		Health center size (m^2^)	508	87	-421	-83%
		OPD visits	8,806	14,780	5,974	68%
		Other health center visits	6,841	11,482	4,641	68%
		Quality of care (%)	47	79	32	
HC 18	0.845348	Personnel cost	41,173	27,681	-13,492	-33%
		Stores	54,880	38,182	-16,698	-30%
		Other expenditure	21,930	8,106	-13,824	-63%
		Health center size (m^2^)	235	172	-63	-27%
		OPD visits	18,401	21,767	3,366	18%
		Other health center visits	16,081	19,023	2,942	18%
		Quality of care (%)	56	66	10	
HC 11	0.972125	Personnel cost	32,368	29,136	-3,232	-10%
		Stores	33,190	32,265	-925	-3%
		Other expenditure	7,013	6,818	-195	-3%
		Health center size (m^2^)	132	128	-4	-3%
		OPD visits	2,982	5,536	2,554	86%
		Other health center visits	17,691	18,198	507	3%
		Quality of care (%)	72	74	2	
HC 14	1	Personnel cost	23,024	21,385	-1,639	-7%
	(CRS TE: 0.924828)	Stores	20,201	17,263	-2,938	-15%
		Other expenditure	3,747	3,747	0	0%
		Health center size (m^2^)	150	122	-28	-19%
		OPD visits	4,726	13,491	8,765	185%
		Other health center visits	5,479	10,488	5,009	91%
		Quality of care (%)	47	47	0	
HC 19	0.654597	Personnel cost	38,732	20,783	-17,949	-46%
		Stores	36,330	23,782	-12,548	-35%
		Other expenditure	25,009	6,576	-18,433	-74%
		Health center size (m^2^)	373	147	-226	-61%
		OPD visits	11,555	17,652	6,097	53%
		Other health center visits	15,234	23,272	8,038	53%
		Quality of care (%)	38	67	29	77%
HC 7	1	Personnel cost	20,335	18,522	-1,813	-9%
	(CRS TE: 0.823031)	Stores	14,649	12,736	-1,913	-9%
		Other expenditure	5,764	3,649	-2,115	-37%
		Health center size (m^2^)	162	104	-58	-36%
		OPD visits	8,851	13,345	4,494	51%
		Other health center visits	2,081	7,060	4,979	239%
		Quality of care (%)	41	41	0	
HC 24	0.893818	Personnel cost	27,394	24,336	-3,058	-11%
		Stores	40,318	34,625	-5,692	-14%
		Other expenditure	8,084	7,038	-1,046	-13%
		Health center size (m^2^)	120	107	-13	-11%
		OPD visits	6,207	8,030	1,823	29%
		Other health center visits	7,393	12,452	5,059	68%
		Quality of care (%)	77	87	9	
HC 22	0.802447	Personnel cost	42,104	28,462	-13,642	-32%
		Stores	52,690	30,517	-22,173	-42%
		Other expenditure	17,521	10,380	-7,141	-41%
		Health center size (m^2^)	492	115	-377	-77%
		OPD visits	6,887	8,582	1,695	25%
		Other health center visits	15,093	22,201	7,108	47%
		Quality of care (%)	70	87	17	
HC 35	0.525233	Personnel cost	35,549	18,672	-16,877	-47%
		Stores	61,822	21,609	-40,213	-65%
		Other expenditure	9,948	5,225	-4,723	-47%
		Health center size (m^2^)	242	110	-132	-54%
		OPD visits	8,895	16,935	8,040	90%
		Other health center visits	12,938	24,633	11,695	90%
		Quality of care (%)	34	66	32	
HCWB 1	0.658735	Personnel cost	58,114	21,060	-37,054	-64%
		Stores	37,100	19,322	-17,778	-48%
		Other expenditure	20,147	4,356	-15,791	-78%
		Health center size (m^2^)	859	97	-762	-89%
		OPD visits	14,396	21,854	7,458	52%
		Other health center visits	5,910	8,972	3,062	52%
		Quality of care (%)	48	73	25	
HCWB 5	0.770264	Personnel cost	72,162	29,787	-42,375	-59%
		Stores	81,684	46,315	-35,369	-43%
		Other expenditure	37,575	11,704	-25,871	-69%
		Health center size (m^2^)	641	210	-431	-67%
		OPD visits	10,059	15,434	5,375	53%
		Other health center visits	24,562	31,888	7,326	30%
		Quality of care (%)	62	81	19	
HCWB 4	0.951985	Personnel cost	61,787	40,456	-21,331	-35%
		Stores Other expenditure	54,937	52,299	-2,638	-5%
		Other expenditure	10,318	9,822	-496	-5%
		Health center size (m^2^)	477	231	-246	-51%
		OPD visits	7,184	9,380	2,196	31%
		Other health center visits	21,834	22,935	1,101	5%
		Quality of care (%)	79	83	4	

*Abbreviations: CRS* Constant returns to scale, *HC* Health center, *HCWB* Health center with beds, *OPD* Outpatient department, *TE* Technical efficiency, *VRS* Variable returns to scale

### Scope of output increases/input reductions to improve efficiency

In total, the avoidable amount of inputs that could have been saved by approaching the efficiency frontier for all 21 VRS inefficient health centers was US$204,491 for staff (US$9,738 per health center), US$241,748 in medical supplies (US$11,512 per health center), and US$112,297 for other operating costs while maintaining the current level of outputs (US$5,347 per health center). Accordingly, the total number of OPD visits would need to increase by 83,407 (+ 18%) and the total number of other contacts by 91,651 (+ 13%) to bring all health centers to the VRS efficiency frontier with the current level of inputs. In addition, quality score improvements of 13% on average could have been achieved if all inefficient VRS inefficient health centers (*n* = 21) had been considered (see Table 4).

### Second stage analysis of the determinants of inefficiency

Table 6 gives the results of the two normal-censored tobit regressions. The CRS inefficiency score was chosen as the dependent variable for model 1 and the VRS inefficiency score for model 2. Both tobit regression models predicting the DEA inefficiency scores from a selection of explanatory variables are statistically significant $\left({Prob>\chi }^{2} =0.003, \mathrm{df }=7\right)$.

**Table 6:** Second stage results from censored tobit regression

**Explanatory variable**	**Model 1**^a^		**Model 2**^**b**^	
	**Censored tobit model CRS**	**t-value**	**Censored tobit model VRS**	**t-value**
OPD visits per year (2016)	- 6.78 *10^–06^	(-0.57)	-8.98*10^–06^	(-0.97)
HC distance to closest provincial referral hospital (km)	0.003	(0.65)	0.001	(0.40)
HC distance to closest referral hospital (km)	0.012	(1.25)	0.018*	(2.41)
Health centers with beds (binary)	0.063	(0.32)	-0.022	(-0.15)
Population coverage by health center (No. of persons)	- 4.36*10^–06^	(-0.29)	-2.78*10^–06^	(-0.24)
Health center size (m^2^)	0.002***	(4.17)	0.001**	(2.80)
Quality Score (%)	-0.009*	(-2.36)	-0.008**	(-2.80)
Constant	0.034	(0.09)	0.155	(0.53)
Sigma	0.30	(0.02)	0.21	(0.04)
**Observations summary**				
Number of observations	43		43	
Log likelihood	-17.81		-8.87	
LR chi2	33.66 (DF = 7)		27.07 (DF = 7)	
Prob > chi2	0.0000		0.0003	
Pseudo R2	0.4859		0.6042	
Left-censored observations	20 (at thetaCRS <= 0)		25 (at thetaVRS <= 0)	
Uncensored observations	20		18	
Right-censored observations	3 (at thetaCRS >= 1)		0	

*Abbreviations: CRS* constant returns to scale, *HC* health center, *OPD* outpatient department, *VRS* variable returns to scale

*p*-value

^*^ *p* < 0.05

^**^ *p* < 0.01

^***^ *p* < 0.001

^a^ Dependent variable representing CRS inefficiency score

^**b**^ Dependent variable representing VRS inefficiency score

In both models the explanatory variables *health center size* and *quality score* are significantly associated with inefficiency. The positive estimate of health center size is significant at the 0.001- level, implying that an increase in building size is inversely correlated with efficiency. The negative coefficient for *quality score* is significant at the 0.05-level, implying that an increase in the quality score results in a decrease of health center inefficiency. In other words: Investing in quality will increase the efficiency of public healthcare facilities in the Cambodian context. Therefore, the assumption that higher quality healthcare would require enormous additional resources so that the efficiency declines, is wrong.

Distance from the health center to the nearest referral hospital (Model 2) was statistically significant at 0.5, implying that longer distances increased inefficiency. A one unit increase in distance to the nearest referral hospital increased technical inefficiency by 0.018, while holding all other variables constant. The second distance variable (distance of health center to the nearest provincial referral hospital) was not significantly associated with inefficiency. The positive coefficient estimate suggests that the inefficiency score increases with distance from the provincial hospital, and thus, from the provincial capital. The number of *OPD visits* *per year *was not significantly associated with inefficiency in both model 1 and 2 nor was the size of *population covered by each health center.*

### Sensitivity analysis

Only minor efficiency changes were observed in our DMUs by examining the variety and diversification of input and output variables. In another robustness testing the six health centers with beds were excluded for DEA score calculations to control for effects on the overall DEA results [46], which produced only minor deviations in the DEA efficiency scores.

## Discussion

This study examined if there is a difference in technical efficiency among a selection of health centers in Cambodia and estimated the magnitudes of output increases and/or input reductions that would have been necessary to increase technical efficiency. In the second stage analysis, coefficient estimates were identified which could help to explain the (preventable) inefficiencies in the Cambodian health centers.

The main study finding of the first stage analysis is that 18 (42%) of 43 of the sample public health centers were not operating on the VRS efficiency frontier. The average VRS efficiency score was 0.93 (*N* = 43) out of a total of 1, scale efficiency score was 0.87 out of a total of 1, and CRS efficiency 0.87 out of a total of 1.

﻿In the Southeast Asian region, only a few studies have applied DEA studies measuring the efficiency on a health facility level. Two DEA studies from the region found an overall efficiency score 0.76 CRS for Vietnamese hospitals [21] and an overall VRS technical efficiency score of 0.82 for public hospitals in Thailand [22]. In the present study, 22 health centers (51%) revealed deficits in producing sufficient outputs from the given amount of inputs at optimal scale size, equaling a 13% efficiency loss (SE = 0.87). Since most health centers operated on increasing returns to scale, the optimal scale size could theoretically be achieved by increasing the number of OPD visits, other patient contacts, and the quality of care in health services.

However, it has been argued that public health facilities have limited control over their outputs, whereby a reduction in inputs is considered more appropriate to optimize efficiency [14]. Less than 20% of the Cambodian population seek care at public health facilities. Even impoverished people - who are entitled to fee waivers - do not necessarily pursue care in these facilities [6, 47]. The public health sector, however, has been forwarded as a means to protect impoverished people in particular from a costly, unregulated and pervasive private health sector [6, 48]. This leads to public efforts to determine attributes that will enable better utilization of public health services [6, 47]. Therefore, this study may provide important information to policymakers and provincial administration that—with the given financial inputs—a 13%-increase in service delivery could be accomplished. For the studied facilities combined, this would translate in an overall annual increase of 83,407 OPD consultations (or 1,940 per facility) and 91,651 other patient contacts (2,131 per health center). In addition, quality score improvements of 13% could have been achieved when compared to high quality health centers in the sample. Nevertheless, there is a goal conflict between equity, efficiency, and costs. Higher efficiency might call for a concentration in fewer health centers. However, this will cause an access problem for the rural population. Achieving one objective (efficiency) might challenge the achievement of the other objective (access for all = equity). Technical efficiency of one institution might not be identical with system efficiency of the entire society and the effectiveness of the entire healthcare system might improve by reducing the technical efficiency of one institution.

The DEA results are reliable benchmarking tools for policy-makers, providing insights that could better inform the allocation of scarce healthcare resources and improve inefficiencies by changing the volume of inputs and/or outputs [38]. According to DEA calculations, the scale-inefficient health centers could have saved US$204,491 in personnel costs, US$241,748 in medical supplies, and US$112,297 in other operating costs. Since measures such as staff reductions or salary cuts are not conducive to sustainable healthcare delivery, reductions to other input-side costs should be considered if quality of care and patient safety can be maintained. In addition, scarce resources, such as personnel, must be used effectively to improve and ensure overall health system performance to match costs. The operational district and health center management therefore have essential roles to play in allocating and balancing the available resources in the best possible way [6]. Therefore, a feasible solution to increase the efficiency of health centers while maintaining high inputs is to increase service utilization and, at the same time, to consistently enforce accountability to separate the blurred lines between the public and private health sectors and habits of dual practice [49]. Health services utilization in Cambodia could be increased by further reducing the barriers and by enrolling more patients, e.g., by attracting patients through outreach activities such as preventive home visits [50]. If utilization of a health center is low, a performance-based bonus system could motivate health workers to make home visits, as repeated visits of economically disadvantaged patients to health providers distant from their homes can impose high economic burden [31]. Many health centers are being provided with motor vehicles. As non-communicable chronic diseases are on the rise in the Southeast Asia region, health centers (including both low and high utilization) may also strengthen their presence through preventative activities by educating schools, businesses and factories about chronic diseases, tobacco and alcohol abuse risks, or promoting healthy diets and physical activity [31, 51].

Ozawa and Walker [52] found that the choice of Cambodian healthcare providers was largely depending on the patient’s trust and on the perceived quality of care as well as other factors such as price and affordability, the availability of medicine, waiting time and receiving intravenous therapy. Additional incentive programs for health check-ups/screenings could further increase utilization and increase trust in public providers, which would affect healthcare-seeking behaviors [52]. Since the shortage of nurses in Cambodia will continuously lead to task-sharing between health workers and family members in the care process, nurses should be trained in strategies to involve the patient’s family. Rather than partially excluding families from the provision of non-medical services, inclusion and recognition of these services could increase citizen acceptance and support for public health centers [53]. As the above examples show, policies can go in different directions to improve the utilization and management of public health services. The key will be to improve the performance, reliability, and quality of care, to remove accessibility barriers, to persuade patients to change their previous behavior in seeking healthcare, and to favor public over private providers [49, 54].

This study showed a significant negative association between better quality health centers and inefficiency in the second stage of the tobit regression. By incorporating the health center quality score as a single independent variable in the second stage sensitivity analysis this showed to have a strong additional effect on the overall efficiency score outcomes. As suggested in previous literature, the incorporation of quality may be a feasible solution to control for the fallacies encountered in many previous health sector efficiency studies that only considered utilization of health services measured by the number of outpatient visits [14, 16, 20, 25, 41]. Quality of care indicators also take into account non-quantifiable aspects of health center performance and could also explain why patient acceptance of health facilities is low and thus services are being avoided [9, 38, 55]. Similarly, Korachais et al. [54] found that both perceived quality of curative services and distance to a health center appeared to be the real barriers to outpatient services utilization in Cambodia. Thus, for the health centers in our sample with both low inefficiency and low-quality scores, health center management should answer the question of how to reduce preventable costs while maintaining or improving quality. To increase both efficiency and quality at low-performing health centers, staff should continuously learn from higher-performing peers and engage in professional training and health workforce management.

The location of more distant and remote health centers could have a critical impact on inefficiency. Results of the second stage tobit regression show that a greater distance of health centers to the adjacent referral hospital was significantly correlated with technical inefficiencies. One reason for this could be that referral hospitals and operational district departments have less control over more distant health centers, so there are fewer routine checks by administrative bodies resulting in worse health center and workforce management [6]. This may also lead to closed health centers during business hours, which would unreasonably increase the patients’ wait time for absent staff to arrive [48].

In this study, we assumed that health centers located in areas closer to urban centers may attract more patients than more remote centers. This is because greater distance to a health facility and residence in rural areas were identified as obstacles for receiving continuous healthcare in antenatal care provision [56]. Karra et al. [57] also found that relatively short distances between patients' homes and healthcare facilities increased service utilization. With regard to healthcare seeking-behavior patterns, many patients may also prefer private over public providers because of the shorter distance from their homes, even if this results in higher out-of-pocket payments for medical treatments [52, 58]. The assumption of greater distance from urban areas as a factor of inefficiency was also echoed by a study from Indonesia by Hafidz et al. [59], which found that the best-performing health facilities are located in more accessible areas that offer better infrastructure for transportation and health facilities. Better transportation and health infrastructure can reduce physical and financial barriers to accessing healthcare for the poor [54, 59]. Another problem that district governments face in rural areas is the difficulty of hiring qualified staff, to ensure the provision of (medical) infrastructure, or controlling staff who engage in dual practices resulting in poor health center performance [49, 56, 59]. This suggests that improving transport and infrastructure in remote areas may possibly attract more qualified staff and also increase accessibility of patients who otherwise would choose another nearby provider [54]. The introduction of a public telemedicine program in Cambodia could be a first starting point, especially considering the local conditions and low-income settings in remote or hard-to-reach areas. Telemedicine also proved particularly useful in many countries that faced contact restrictions during the Covid-19 pandemic [60, 61].

One other factor for technical efficiency measurement was health center size (m^2^). We found several larger-in-size health centers with and without beds. Particularly for the inefficient health center with beds, reducing the size of the building could improve their efficiency scores, as building size often does not match the overall output of health services. Similarly, in a hospital study from Ghana, Juhu-Appiah et al. [25] recommended that downsizing a hospital building below a certain threshold should be considered if the inefficiency of the healthcare facility is related to a large building size. These findings are also consistent with the study’s coefficient estimates that an increase in building size is associated with health center inefficiency.

The coefficient number of OPD visits per healthcenter as a measure of health care utilization was not significantly associated with the dependent variable. Also, the coefficient population coverage per health center as a measure of size of the catchment area per health facility did not show a significant relationship. The coefficient was included to examine if the effect of catchment areas with lower and higher population coverage determines inefficiency of a health center. However, as Hafidz et al. [59] shows, population density may be a more important factor than the number of people covered by a healthcare facility.

### Limitations

Despite the numerous findings, the study has some limitations to be considered when interpreting the results. First, the data used in this analysis is from 2016–2017. This is because no further costing study on health center level has been carried out. It is known to the authors of this research that a national costing of health centers was repeated in 2019, but these results were not published. Consequently, the authors had to rely on the older data. Since 2017, the service delivery grants (SDGs) were introduced in Cambodia which provide more direct financial resources to public health facilities. This is a limitation of the findings of this paper. However, unofficial communication about the findings of the 2019 data made it clear that SDGs have a very limited budget and do not alter the costing results significantly. Thus, the data used in this paper is still representative for the Cambodian situation of health center costing.

Second, we did not simulate different DEA models, although there would be other alternatives, such as Directional Distance Functions or other inputs/outputs. The results are rather convincing so that amendments of the principal assumptions of the paper did not seem necessary. However, future research might focus more on the methodological issues and simulate different models to see the impact of model changes on results. In this paper, we focussed on the learnings for Cambodia’s leadership.

Third, DEA should only be applied to enable relative comparisons of a particular set of peer units, as the DEA first stage does not provide any empirically proven method for measuring efficiency. This makes it challenging to generalize findings and therefore DEA results should be interpreted with caution. The DEA values only reflect a specific set of economic factors in a certain time frame and completely mask possible individual factors such as regional circumstances [24]. Many studies, including this one, therefore try to heal this shortcoming by conducting a second stage regression analysis [38, 39].

Fourth, even though sensitivity analyses were employed in this study, the effect of ‘noise’ in the data cannot be fully ruled out for DEA results [24]. Using a relatively small sample size, some of the input and output variables had large ranges of standard deviations and outlier values.

The inclusion of the number of other patient contacts as a variable was a necessary concession to perform DEA calculations using a second health service utilization variable in addition to the outpatient department visits, even though undesirable missing values were knowingly included for some observations. This limitation should raise caution about the internal validity and generalizability of this variable, as its impact on efficiency may be underestimated for some of the DMUs with missing data. Other studies sought to address external validity using time-series based DEA methods by processing panel data capable of measuring efficiency changes over time [15, 16]. In this way, implausibility in the data could be identified. However, this approach is highly dependent on the future availability and accuracy of time series data for Cambodia.

## Conclusion

This study found that there is a wide scope for improving the efficiency of public health centers in Cambodia. Cambodia’s public health strategy targets the provision of effective and quality health services. An important step towards achieving universal health coverage and achieving Cambodia’s public health strategy is therefore to more rationally allocate health center resources and increase cost effectiveness, to address health workforce shortages, and to ensure sustainable public health financing. In this study, a significant difference in relative technical efficiency was found in a sample of 43 public health centers in Cambodia. A total of 41% of the health centers were classified as technically inefficient facilities (with variable returns to scale) that could increase their productive capacity, including the quality of care, by a total of 13%. This study identified greater distances between health centers and the nearest referral hospitals, health center size, and low quality of health center services as potential predictors of preventable inefficiencies. To address this, (technically) inefficient health centers must improve their healthcare provision and quality levels with the given amount of input resources. By planning for adjustments in all these determinants, unclaimed resources could be made available to care for additional patients in need.

Our analysis clearly shows that poor utilization in some health centers is the main cause of technical inefficiency of these institutions. Overall, there is more than enough demand for healthcare services to increase utilization, but many people perceive the quality of public healthcare as low and therefore avoid these services. Consequently, the utilization and the technical efficiency are low. This problem can only be addressed by better quality of services, including structural (e.g., more and better-trained personnel, friendliness of staff, improved buildings, availability of equipment and drugs), process (e.g., lower waiting times) and results (e.g., treatment success) dimensions of quality. This investment in improved quality will require additional resource management, but as Jacobs et al. [33] have shown, the majority of these costs are fixed, i.e., the additional investment will pay-off and the technical efficiency will increase by investing in quality of services.

Consequently, the technical efficiency of the public health system of Cambodia will strongly improve if the Royal Government of Cambodia strengthens its efforts to improve quality of services, organizational and health center management. This includes a continuous monitoring of health center performance over time.

Finally, this study highlights the lack of health economic research in Cambodia. We would have preferred to build our analysis on more recent data, but the 2016/17 data are the latest available on health center-specific costing. It is a tremendous challenge that health policy is frequently based on obsolete data, particularly in costing. Therefore, there is a need to establish a routine costing system for healthcare services in Cambodia. At the same time, data approval processes within the government system need to be expedited so that government health policies are more evidence-based.

## Data Availability

The data that support the findings of this study were used under license from Deutsche Gesellschaft für Internationale Zusammenarbeit GmbH (GIZ), and are not publicly available. Data is however available from the corresponding author upon reasonable request and with permission of GIZ.

## References

[1] Ensor T, Chhun C, Kimsun T, McPake B, Edoka I (2017). Impact of health financing policies in Cambodia: A 20 year experience. Soc Sci Med.

[2] World Health Organization Regional Office for the Western Pacific. Cambodia – WHO country cooperation strategy 2016–2020; Manila, Philippines; 2016.

[3] World Health Organization. Health Service Delivery Profile Cambodia 2012. 2012.

[4] Marmot M (2015). The health gap : the challenge of an unequal world.

[5] Chua AQ, Tan MMJ, Verma M, Han EKL, Hsu LY, Cook AR (2020). Health system resilience in managing the COVID-19 pandemic: lessons from Singapore. BMJ Glob Heal.

[6] Ministry of Health Cambodia. The Third Health Strategic Plan 2016–2020 (HSP3) Quality, Effective and Equitable Health. Phnom Penh; 2016.

[7] World Health Organization, Yip W, Hafez R. Improving health system efficiency: reforms for improving the efficiency of health systems: lessons from 10 country cases. World Health Organization; 2015.

[8] World Health Organization. The World Health Report 2000: Health Systems. Geneva: Improving performance; 2000.

[9] Marschall P, Flessa S. Efficiency of primary care in rural Burkina Faso. A two-stage DEA analysis. Heal Econ Rev. 2011;1(1):5. 10.1186/2191-1991-1-5. 10.1186/2191-1991-1-5 PMC3395044 22828358

[10] Farrell M (1957). The measurement of productive efficiency. J R Stat Soc.

[11] Watcharasriroj B, Tang JCS (2004). The effects of size and information technology on hospital efficiency. J High Technol Manag Res.

[12] World Health Organization (2015). The Kingdom of Cambodia Health System Review.

[13] Farrell MJ (1957). The measurement of productive efficiency. J R Stat Soc Ser A.

[14] Cantor VJM, Poh KL (2018). Integrated analysis of healthcare efficiency: a systematic review. J Med Syst.

[15] Ensor T, So S, Witter S (2016). Exploring the influence of context and policy on health district productivity in Cambodia. Cost Eff Resour Alloc.

[16] Kohl S, Schoenfelder J, Fügener A, Brunner JO. The use of Data Envelopment Analysis (DEA) in healthcare with a focus on hospitals. Health Care Manag Sci. 2019;22(2):245-86. 10.1007/s10729-018-9436-8. Epub 2018 Feb 24. Erratum in: Health Care Manag Sci. 2018 Mar 29; PMID: 29478088. 10.1007/s10729-018-9436-8 29478088

[17] Kolesar RJ, Bogetoft P, Chea V, Erreygers G, Pheakdey S (2022). Advancing universal health coverage in the COVID-19 era: an assessment of public health services technical efficiency and applied cost allocation in Cambodia. Health Econ Rev.

[18] Halkos GE, Tzeremes NG (2010). A conditional nonparametric analysis for measuring the efficiency of regional public healthcare delivery: an application to Greek prefectures. Health Policy (New York).

[19] Araújo C, Barros CP, Wanke P (2014). Efficiency determinants and capacity issues in Brazilian for-profit hospitals. Health Care Manag Sci.

[20] Kirigia JM, Asbu EZ (2013). Technical and scale efficiency of public community hospitals in Eritrea: an exploratory study. Health Econ Rev.

[21] Pham TL (2011). Efficiency and productivity of hospitals in Vietnam. J Heal Organ Manag An Int J Iss An Int J.

[22] Puenpatom RA, Rosenman R (2008). Efficiency of Thai provincial public hospitals during the introduction of universal health coverage using capitation. Health Care Manag Sci.

[23] Tiemann O, Schreyögg J, Busse R (2012). Hospital ownership and efficiency: a review of studies with particular focus on Germany. Health Policy (New York).

[24] Akazili J, Adjuik M, Jehu-Appiah C, Zere E (2008). Using data envelopment analysis to measure the extent of technical efficiency of public health centres in Ghana. BMC Int Health Hum Rights.

[25] Jehu-Appiah C, Sekidde S, Adjuik M, Akazili J, Almeida SD, Nyonator F (2014). Ownership and technical efficiency of hospitals: evidence from Ghana using data envelopment analysis. Cost Eff Resour Alloc.

[26] Novignon J, Nonvignon J (2017). Improving primary health care facility performance in Ghana: efficiency analysis and fiscal space implications. BMC Health Serv Res.

[27] Sebastian MS, Lemma H (2010). Efficiency of the health extension programme in Tigray, Ethiopia: a data envelopment analysis. BMC Int Health Hum Rights.

[28] Marschall P, Flessa S (2009). Assessing the efficiency of rural health centres in Burkina Faso: an application of data envelopment analysis. J Public Health (Bangkok).

[29] World Bank Group. Cambodia - Sustaining strong growth for the benefit of all: a systematic country diagnostic (English). Washington D.C.; 2017. http://documents.worldbank.org/curated/en/620151496155751423/Cambodia-Sustaining-strong-growth-for-the-benefit-of-all-a-systematic-country-diagnostic. Accessed 6 Aug 2023.

[30] Ministry of Planning Cambodia. General Population Census of the Kingdom of Cambodia 2019: National Report on Final Census Results. 2020. https://www.nis.gov.kh/nis/Census2019/Final%20General%20Population%20Census%202019-English.pdf. Accessed 6 Aug 2023.

[31] Jacobs B, Hill P, Bigdeli M, Men C (2016). Managing non-communicable diseases at health district level in Cambodia: a systems analysis and suggestions for improvement. BMC Health Serv Res.

[32] Ministry of Health Cambodia. Guidelines For the Benefit Package and Provider Payment of the Health Equity Fund for the Poor. Phnom Penh; 2018. https://moh.gov.kh/content/uploads/2017/05/Guideline-BP-English.pdf. Accessed 6 Aug 2023.

[33] Jacobs B, Hui K, Lo V, Thiede M, Appelt B, Flessa S (2019). Costing for universal health coverage: Insight into essential economic data from three provinces in Cambodia. Health Econ Rev.

[34] Fritsche G, Peabody J (2018). Methods to improve quality performance at scale in lower- and middle-income countries. J Glob Health.

[35] Open Development Cambodia. OpenDevelopment Cambodia Map-explorer. Map explorer. 2018. https://opendevelopmentcambodia.net/map-explorer. Accessed 3 Apr 2018.

[36] Cooper W, Seiford L, Zhu J. Handbook on Data Envelopment Analysis. 2011. 10.1007/978-1-4419-6151-8.

[37] Bogetoft P (2012). Performance Benchmarking.

[38] Ozcan YA (2014). Health Care Benchmarking and Performance Evaluation, An Assessment using Data Envelopment Analysis (DEA).

[39] Coelli T, Rao P, O'Donnell CJ, Battese GE. An Introduction to Efficiency and Productivity Analysis. Springer; 2005. 10.1007/b136381.

[40] Charnes A, Cooper WW, Rhodes E (1978). Measuring the efficiency of decision making units. Eur J Oper Res.

[41] Mujasi PN, Asbu EZ, Puig-Junoy J (2016). How efficient are referral hospitals in Uganda? A data envelopment analysis and tobit regression approach. BMC Health Serv Res.

[42] Valdmanis V (1992). Sensitivity analysis for DEA models: An empirical example using public vs. NFP hospitals J Public Econ.

[43] Cooper Z, Gibbons S, Jones S, McGuire A (2011). Does hospital competition save lives? Evidence from the English NHS patient choice reforms. Econ J (London).

[44] Fixsen D, Naoom S, Friedman R, Wallace F. Implementation Research: A Synthesis of the Literature. 231st edition. FHMI Publication; 2005.

[45] Ji Y-B, Lee C (2010). Data envelopment analysis in stata. Stata J.

[46] Ozcan YA (1992). Sensitivity analysis of hospital efficiency under alternative output/input and peer groups: a. Knowl Policy.

[47] Asante AD, Ir P, Jacobs B, Supon L, Liverani M, Hayen A (2019). Who benefits from healthcare spending in Cambodia? Evidence for a universal health coverage policy. Health Policy Plan.

[48] Itay N, Saing H. Determinants of Non-Utilisation of Public Health Services among Poor Households Covered by a Social Health Protection Scheme, An Evaluation in Kampot Operational District, Cambodia. Dtsch Gesellschaft für Int Zusammenarbeit. 2012.

[49] Gryseels C, Kuijpers LMF, Jacobs J, Peeters GK (2019). When ‘substandard’ is the standard, who decides what is appropriate? Exploring healthcare provision in Cambodia. Crit Public Health.

[50] Kovacs RJ, Powell-Jackson T, Kristensen SR, Singh N, Borghi J (2020). How are pay-for-performance schemes in healthcare designed in low- and middle-income countries? Typology and systematic literature review. BMC Health Serv Res.

[51] Dans A, Ng N, Varghese C, Tai ES, Firestone R, Bonita R (2011). The rise of chronic non-communicable diseases in southeast Asia: time for action. Lancet.

[52] Ozawa S, Walker DG (2011). Comparison of trust in public vs private health care providers in rural Cambodia. Health Policy Plan.

[53] Sakurai-Doi Y, Mochizuki N, Phuong K, Sung C, Visoth P, Sriv B (2014). Who provides nursing services in Cambodian hospitals?. Int J Nurs Pract.

[54] Korachais C, Ir P, Macouillard E, Meessen B (2019). The impact of reimbursed user fee exemption of health centre outpatient consultations for the poor in pluralistic health systems: lessons from a quasi-experiment in two rural health districts in Cambodia. Health Policy Plan.

[55] Chan Soeung S, Grundy J, Duncan R, Thor R, Bilous JB (2013). From reaching every district to reaching every community: analysis and response to the challenge of equity in immunization in Cambodia. Health Policy Plan.

[56] Yasuoka J, Nanishi K, Kikuchi K, Suzuki S, Ly P, Thavrin B (2018). Barriers for pregnant women living in rural, agricultural villages to accessing antenatal care in Cambodia: a community-based cross-sectional study combined with a geographic information system. PLoS ONE.

[57] Karra M, Fink G, Canning D (2017). Facility distance and child mortality: a multi-country study of health facility access, service utilization, and child health outcomes. Int J Epidemiol.

[58] Jacobs B (2004). The impact of the introduction of user fees at a district hospital in Cambodia. Health Policy Plan.

[59] Hafidz F, Ensor T, Tubeuf S (2018). Assessing health facility performance in Indonesia using the Pabón-Lasso model and unit cost analysis of health services. Int J Health Plann Manage.

[60] Intan Sabrina M, Defi IR (2021). Telemedicine guidelines in South East Asia—a scoping review. Front Neurol.

[61] Nit B, Kobashi Y, Vory S, Lim S, Chea S, Ito S (2021). The introduction of telemedicine is required immediately in Cambodia: barriers and lessons from COVID-19. J Glob Health.

